# Risk factors, short and long term outcome of anastomotic leaks in rectal cancer

**DOI:** 10.18632/oncotarget.5170

**Published:** 2015-09-16

**Authors:** Olof Jannasch, Tim Klinge, Ronny Otto, Costanza Chiapponi, Andrej Udelnow, Hans Lippert, Christiane J. Bruns, Pawel Mroczkowski

**Affiliations:** ^1^ Department for General, Visceral and Vascular Surgery, Otto-von-Guericke-University, Magdeburg, Germany; ^2^ Department for General and Abdominal Surgery, AMEOS Hospital, Haldensleben, Germany; ^3^ Institute for Quality Assurance in Operative Medicine, Otto-von-Guericke-University, Magdeburg, Germany

**Keywords:** quality assurance, rectal cancer, anastomotic leak, short term outcome, long term outcome

## Abstract

**Background:**

An anastomotic leak (AL) after colorectal surgery is one major reason for postoperative morbidity and mortality. There is growing evidence that AL affects short and long term outcome. This prospective German multicentre study aims to identify risk factors for AL and quantify effects on short and long term course after rectal cancer surgery.

**Methods:**

From 1 January 2000 to 31 December 2010 381 hospitals attributed patients to the prospective multicentre study Quality Assurance in Colorectal Cancer managed by the Otto-von-Guericke-University Magdeburg (Germany). Included were 17 867 patients with histopathologically confirmed rectal carcinoma and primary anastomosis. Risk factor analysis included 13 items of demographic patient data, surgical course, hospital volume und tumour stage.

**Results:**

In 2 134 (11.9%) patients an AL was diagnosed. Overall hospital mortality was 2.1% (with AL 7.5%, without AL 1.4%; *p* < 0.0001). In multivariate analysis male gender, ASA-classification ≥III, smoking history, alcohol history, intraoperative blood transfusion, no protective ileostomy, UICC-stage and height of tumour were independent risk factors. Overall survival (OS) was significantly shorter for patients with AL (UICC I-III; UICC I, II or III - each *p* < 0.0001). Disease free survival (DFS) was significantly shorter for patients with AL in UICC I-III; UICC II or UICC III (each *p* < 0.001). Rate of local relapse was not significantly affected by occurrence of AL.

**Conclusion:**

In this study patients with AL had a significantly worse OS. This was mainly due to an increased in hospital mortality. DFS was also negatively affected by AL whereas local relapse was not. This emphasizes the importance of successful treatment of AL related problems during the initial hospital stay.

## INTRODUCTION

Colorectal cancer is the second most common cause of tumour-related death in Europe[1]. An anastomotic leak (AL) is a major reason for postoperative morbidity and mortality as well as reduced quality of life [2, 3]. Frequency of AL after rectal cancer surgery ranges from 2.6% to 19.0%[3–10]. Several risk factors affecting the healing of the colorectal anastomosis have been identified [6–8, 11]. In particular, healing of colorectal anastomosis might be affected by amount of intraoperative blood loss, tumour height (ultra-low anterior resection) and the surgeon. AL is also related to prolonged stay in hospital and increased treatment costs [1, 7, 12, 13]. Furthermore, there is growing evidence that AL effects short and long term survival and frequency of tumour relapse [14, 15]. Based on the data of the multicentre International Quality Assessment Study in Colorectal Cancer [16] we looked for risk factors and consequences of AL in surgery of rectal cancer.

## RESULTS

From 1 January 2000 to 31 December 2010 a total of 17 867 patients from 381 hospitals fulfilled the inclusion criteria. Detailed patient characteristics are given in Table 1. AL occurred in 2 134 (11.9%) cases. Hospital mortality was 2.1% and demonstrated significant difference between patients with (7.5%) and without (1.4%) AL (*p* < 0.0001).

**Table 1:** Detailed patient characteristics

	Total	patients with AL	patients without AL	*p*
**patients *n* (%)**	17 867 (100%)	2 134 (11.9%)	15 733 (88.1%)	<0.0001*
**gender *n* (%)**	17 827 (100%)	2 127 (11.9%)	15 700 (88.1%)	<0.0001*
**(ratio - male:female)**	1.4:1	2.5:1	1.3:1	
**mean age (years ± SD)**	66.95 ± 10.54	66.31 ± 10.24	67.03 ± 10.57	<0.0001**
**mean body mass index (kg/m^2^ ± SD)**	26.49 ± 4.22	26.54 ± 4.24	26.49 ± 4.21	0.485**
**mean stay in hospital (days ± SD)**	21 ± 12.7	37 ± 19.9	19 ± 9.8	<0.0001**
**hospital mortality *n* (%)**	379 (2.1%)	160 (7.5%)	219 (1.4%)	<0.0001*

* Chi-square test

** Mann-Whitney-*U*-test

AL - anastomotic leakage

Univariate analysis revealed gender, ASA-classification, smoking, alcohol, intraoperative blood transfusion, no protective ileostomy, UICC-stage and height of tumour as significant risk factors. Results are given in Table 2. Gender, ASA-classification, smoking, alcohol, intraoperative blood transfusion, no protective ileostomy, UICC-stage and height of tumour remained significant in multivariate analysis. Results are given in Table 3.

**Table 2:** Risk factors for anastomotic leakage - univariate analysis

	*n*	anastomotic leakage *n* (%)	no anastomotic leakage *n* (%)	*p*
**male**	10 477	1 524 (14.5%)	8 953 (85.5%)	**<0.0001**
**female**	7 350	603 (8.2%)	6 747 (91.8%)	
**age ≤68 years**	9 592	1 170 (12.2%)	8 422 (87.8%)	0.261
**age >68 years**	8 266	963 (11.7%)	7 303 (88.3%)	
**BMI ≤25 kg/m^2^**	8 513	1 010 (11.9%)	7 503 (88.1%)	0.945
**BMI >25 kg/m^2^**	8 707	1 036 (11.9%)	7 671 (88.1%)	
**ASA I and II**	11 733	1 314 (11.2%)	10 419 (88.8%)	**<0.0001**
**ASA III and IV**	6 107	818 (13.4%)	5 289 (86.6%)	
**smoking yes**	954	165 (17.3%)	789 (82.7%)	**<0.0001**
**smoking no**	16 804	1 953 (11.6%)	14 851 (88.4%)	
**alcohol yes**	347	74 (21.3%)	273 (78.7%)	**<0.0001**
**alcohol no**	17 411	2 044 (11.7%)	15 367 (88.3%)	
**diabetes mellitus yes**	1 114	140 (12.6%)	974 (87.4%)	0.496
**diabetes mellitus no**	16 644	1 978 (11.9%)	14 666 (88.1%)	
**hospital volume < 14ppy**	4 632	541 (11.7%)	4 091 (88.3%)	0.314
**hospital volume 15–24 ppy**	4 737	543 (11.5%)	4 194 (88.5%)	
**hospital volume 25–36 ppy**	4 213	544 (12.1%)	3 669 (87.9%)	
**hospital volume > 36 ppy**	3 985	506 (12.7%)	3 479 (87.3%)	
**stapled anastomosis**	16 612	1 983 (11.9%)	14 629 (88.1%)	0.721
**hand sutured anastomosis**	1 046	121 (11.6%)	925 (88.4%)	
**blood transfusion yes**	228	39 (17.1%)	189 (82.9%)	**0.016**
**blood transfusion no**	17 639	2 095 (11.9%)	15 544 (88.1%)	
**ileostomy yes**	7 148	893 (12.5%)	6 255 (87.5%)	**0.003**
**ileostomy no**	5 057	724 (14.3%)	4 333 (85.7%)	
**UICC I**	6 457	721 (11.2%)	5 736 (88.8%)	**0.004**
**UICC II**	5 162	600 (11.6%)	4 562 (88.4%)	
**UICC III**	6 248	813 (13.0%)	5 435 (87.0%)	
**tumour heigth < 6 cm**	1 703	256 (15.0%)	1 447 (85.0%)	**<0.0001**
**tumour heigth 6–12 cm**	11 144	1 421 (12.8%)	9 723 (87.2%)	
**tumour heigth > 12 cm**	4 501	400 (8.9%)	4 101 (91.1%)	

p for Chi-square test, ASA - classification of the American Society of Anaesthesiologists, ppy - patients per year

**Table 3:** Risk factors for anastomotic leakage - multivariate analysis

	*p*	odds ratio	95% CI	
**tumour height >12 cm**		1		
**tumour height 6–12 cm**	**<0.0001**	2.120	1.765	2.546
**tumour height <6 cm**	**<0.0001**	1.619	1.428	1.835
**male**	**<0.0001**	1.923	1.733	2.133
**intraop. blood transfusion**	**0.024**	1.510	1.055	2.160
**UICC I**		1		
**UICC II**	0.724	1.022	0.906	1.152
**UICC III**	**<0.0001**	1.228	1.100	1.372
**smoking**	**0.002**	1.332	1.106	1.604
**no ileostomy**	**<0.0001**	1.222	1.105	1.351
**alcohol**	**0.001**	1.628	1.233	2.150
**ASA-classification III + IV**	**<0.0001**	1.214	1.101	1.338

CI - confidence interval

**Figure 1:**
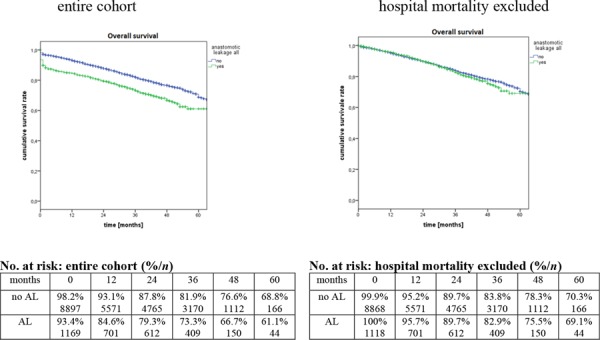
Probability of 5-year overall survival

Median follow-up was 30 months and included 79.9% of patients, who gave consent for data collection (81% of the entire cohort). Patients with AL had a lower probability of the OS. The difference was significant for the whole cohort (*p* < 0.0001) as well as for the subgroups (UICC I, II, III - each *p* < 0.0001). Further analysis revealed that this difference originates from in hospital death. Whereas probability of overall survival differed significantly for the entire cohort, no significant difference could be shown for both groups, if patients who died during the hospital stay were excluded. Detailed data are shown in Figure 1 as well as in Table 4.

**Table 4:** Probability of 5-year overall survival according to tumour stage and occurrence of anastomotic leak

tumour stage	5y-OS				5y-OS - without hospital mortality			
	total	AL yes	AL no	*p*	total	AL yes	AL no	*p*
**UICC I-III**	67.8%	61.1%	68.8%	**<0.0001**	70.2%	69.1%	70.3%	0.339
**UICC I**	80.0%	78.8%	80.1%	**<0.0001**	82.2%	85.8%	81.7%	0.913
**UICC II**	66.5%	59.9%	67.3%	**<0.0001**	69.0%	68.5%	68.9%	0.837
**UICC III**	56.6%	46.9%	58.2%	**<0.0001**	58.9%	54.3%	59.5%	0.764

*p* for Chi-square test, AL - anastomotic leakage, OS - overall survival

**Figure 2:**
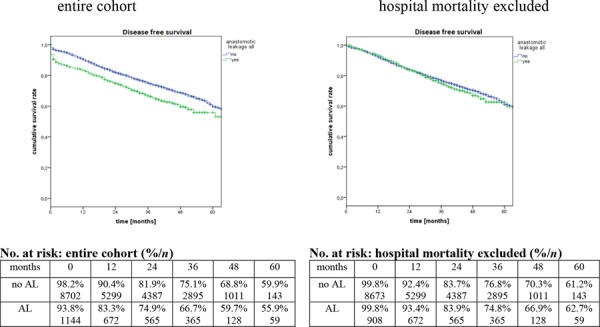
Probability of 5-year disease free survival

DFS was also effected by AL. The difference was significant for the whole cohort (*p* < 0.0001) as well as for the subgroups (UICC I – *p* = 0.005; UICC II – *p* = 0.001; UICC III – *p* < 0.0001). Patients with AL and UICC I displayed an increased 5-year DFS. Otherwise, patients with UICC II, UICC III or the whole cohort had an decreased DFS. As for OS no group difference could be shown, if patients who died during the hospital stay were excluded. Detailed data are shown in Figure 2 as well as in Table 5.

**Table 5:** Probability of disease free survival according to tumour stage and occurrence of an anastomotic leak

tumour stage	5-y DFS				5y-DFS - without hospital mortality			
	total	AL yes	AL no	*p*	total	AL yes	AL no	*p*
**UICC I-III**	59.4%	55.9%	59.9%	**<0.0001**	61.4%	62.7%	61.2%	0.389
**UICC I**	73.9%	75.1%	73.6%	**0.005**	95.9%	81.8%	75.1%	0.769
**UICC II**	57.1%	55.9%	75.0%	**0.001**	59.2%	63.7%	58.4%	0.553
**UICC III**	46.3%	38.9%	47.5%	**<0.0001**	48.0%	44.2%	48.5%	0.268

*p* for Chi-square test, AL - anastomotic leakage, DFS - disease free survival

AL had no significant effect on the probability of local relapse (UICC I-III *p* = 0.240, UICC I *p* = 0.671, UICC II *p* = 0.376, UICC III *p* = 0.704) as demonstrated in Table 6.

**Table 6:** Probability of a local relapse according to tumour stage and occurrence of an anastomotic leakage

Local relapse	total	AL yes	AL no	*p*
**UICC I-III**	9.3%	8.3%	9.4%	0.240
**UICC I**	5.4%	4.6%	5.5%	0.671
**UICC II**	8.2%	8.0%	8.4%	0.376
**UICC III**	15.2%	12.1%	15.7%	0.704

*p* for Chi-square test, AL - anastomotic leakage

## DISCUSSION

The observed AL rate of 11.9% fits in the range from 2.7 to 19% described in other studies [5, 10, 13, 20–26]. Multivariate analysis displayed male gender, smoking, alcohol use, UICC-stage III, ASA-classification III+IV, intraoperative blood transfusion, no protective ileostomy, tumour localisation in the middle and lower rectum as independent risk factors for AL.

### Patient-related factors

Male gender was accompanied by a 1.7 fold risk for AL. This was confirmed by other studies (OR 1.49 - 2.36) [5, 10, 13, 24] and might be attributed to the anatomical difference in comparison to the wider female pelvis. Furthermore use of alcohol and smoking is more common in male. The well-known negative effect on general wound healing[5, 26] might explain that smoking history was an independent risk factor for AL in this study (OR 1.3). Others estimated the negative effect even higher [27]. Richards et al. found on multivariate analysis that current smokers have a significantly increased risk for AL undergoing low anterior resection (OR 3.68, 95% CI 1.38–9.82, *p* = 0.009) [6]. Even though alcohol history was associated with a higher risk for AL than smoking (OR 1.6 vs. 1.3) a generally negative effect on wound healing is not proven. A limiting factor for analysing the effect of alcohol and smoking is the reliability of the patients report.

ASA- classification III or IV has been identified as risk factor for wound healing disturbances [10, 28]. However, Bertelsen et al. did not find a significant correlation between ASA-classification and AL, but in this study only ASA-score I and II were included [5]. Chemo- or radiochemotherapy (neo-adjuvant or adjuvant) might alter risk of anastomotic leak [10, 29]. This risk factor was not included in the current analysis. Particularly neo-adjuvant treatment was no standard treatment in the entire cohort.

### Tumour-related factors

In comparison to UICC stage I – stage II does not increase risk for AL. This is consistent with findings of other authors[30]. Smith analyzed the effect of UICC stage in general on rate of AL and did not find a statistical significant correlation (*p* = 0.15)[21]. However, in a bootstrap analysis Warschkow et al. identified UICC stage III or IV as risk factor for AL[22]. Bertelsen et al. reported no statistical influence of tumour stage on risk of AL[5]. The classification of the tumour stage highly depends on the assessment of the specimen by the local pathologist and might be a subject of interobserver variation[31].

Several studies reported a correlation between tumour height and rate of AL. Highest risk was found for anastomosis in the lower rectum[5, 22, 23]. In the current study we found the highest risk for anastomoses in the middle rectum (OR 2.2 for middle rectum vs. OR 1.8 for lower rectum). A speculative explanation could be selection of more experienced surgeons for performing a lower resection and anastomosis, but our data do not deliver this information. Otherwise, a currently published systematic review by McDermott et al. states that for rectal procedures the distance from the anal margin is a significant predictor of AL, with the risk increasing the closer the tumour is to the anal margin[10].

### Surgery-related factors

Hospital-volume did not show significant impact even in the univariate analysis. The discussion about the role of the hospital-volume lasts for nearly 20 years and is mostly supported by American data[32] with limited reproduction in Europe. It is possible that a volume-effect is not present or far reduced in hospitals participating in quality-assurance programs, as reported by our group for colon cancer [17] and recently also from the Belgian PROCARE programme [33].

Intraoperative blood transfusion has been identified as risk factor for AL [5, 10, 22]. It is also known as a risk factor for wound healing disturbances in general [34]. However, there seems to be an effect of the amount of blood given during operation [22, 35]. In the current study we found a 1.5-fold risk for AL without differentiation of the amount of blood units given. The possible biologic mechanisms of the worse outcomes were widely discussed before [36], but in any case blood loss is a proxy for surgical quality and subtle preparation technique. Protective ileostomy has been identified as effective procedure to reduce risk of AL [5, 21–23, 37–39], also in a recently published Indian randomized controlled trial [40]. This is supported by the finding of the current study. Lack of an ileostomy increased the risk for an AL (OR 1.2), so we also support faecal diversion for reduction of AL. Since we have no data about the percentage of low anastomosis vs. anastomosis in the mid-rectum the relation of ileostomy and anastomotic leak is correlated with tumour height. However, creation of a stoma might only lessen the consequences but not the prevalence of AL [10].

### Longterm results

The current study could demonstrate a significant negative effect of AL on OS in rectal cancer surgery. This can mainly be attributed to the in hospital mortality in the postoperative course, probably resulting from septic problems. Tumour biology does not seem to be affected, as the OS of AL survivors does not differ. Bertelsen et al. demonstrated a 4-fold 30-days-mortality in case of AL in a multicentre study including 1 494 patients with rectal cancer [4]. A meta-analysis by Mirnezami et al. demonstrated a significantly higher specific long term cancer mortality after AL (OR 1.75; 95% CI = 1.47–2.1; *p* = 0.0001) [41]. But this analysis is based on studies published between 1965–2009. Even though it comprises data of 21902 patients from 21 studies only 1 prospective randomized study is included. Otherwise, Mrak et al. did not find a correlation between AL and OS in a unicentre study with 811 patients [42]. Even though number of included patients is small in that study, follow up was 20 years. A follow up of the Swedish rectal cancer registry from 1995–1997 did also not show a correlation between AL and OS but also demonstrated a higher 30-days -mortality of patients with AL [25]. In conclusion, it seems as if the patient survives an AL he may not experience disadvantages concerning his OS. It also implies that patients having multiple risk factors for AL should be treated in specialized centres.

We could demonstrate a statistical significant effect of AL on DFS (*p* < 0.0001) but not for local relapse (*p* = 0.958). Otherwise, DFS did not differ between groups if in hospital mortality was excluded. So again tumour biology does not seem to be affected, in survivors of AL. This correlates with other findings. Mrak et al. and Smith et al. did also not find a correlation between AL and either DFS or local relapse [21, 42]. Multivariate analyses of the Danish national Register did not show an increase of local and distant recurrence in patients with AL after anterior resection for rectal cancer [4]. Ptok et al. could demonstrate a higher 5-year local relapse rate (4.3 vs. 1.2%, *p* = 0.006) for patients with AL necessitating surgical treatment [43]. But currently interventional drainage of abscesses is largely available und reduces rate of reoperation. Mirzenami et al. could also demonstrate an increased risk for developing a local relapse after rectal anastomoses (OR 2.05; 95% CI = 1.51–2.8; *p* = 0.0001) [41]. But significant heterogeneity was detected in the local recurrence outcomes, which may indicate that the data was not suitable for pooling.

According to Jörgren et al. DFS seems to be affected during the first few years but not so in the long term course [25]. Differences between findings may also result from different definitions of AL. Jörgren et al. defined AL by “symptoms or clinical signs” [25] whereas Bertelsen et al. provide a detailed definition: “AL was defined as follows: peritonitis and a defect in the anastomosis, discharge of pus from the anus, and recto-vaginal fistula or faeces or gas from the abdominal drain. Leakage was confirmed by endoscopy, CT scan, contrast enema, reoperation or digital rectal examination” [4]. Efforts to unify classification of anastomotic leakage are under progress, but an implementation is a future question [20].

A limitation of our study is the quality of the follow-up data. Even though there is an improvement in cancer documentation in Germany[44], the reporting is based mostly on family physician's goodwill. This voluntary programme without public support could not compare the documented data with clinical reality.

## MATERIALS AND METHODS

### Study design and data assessment

The analysis concerned patients with cancer of the rectum treated from 1 January 2000 to 31 December 2010 and recorded in the prospective International Quality Assessment Study in Colorectal Cancer managed by the Otto-von Guericke-University Magdeburg (Germany). The project was voluntary and based on the anonymity of both patients and hospitals. 381 of 1100 German hospitals performing anterior resection of the rectum could be included in this study. Data were provided by surgical departments for every patient treated for colorectal cancer and documented in a structured questionnaire by the attending surgeon. Included were all patients with histopathologically confirmed rectal carcinoma and primary anastomosis. The hospitals were required to deliver data on every patient treated for rectal cancer and the total number of reported patients was cross-checked with the hospital's financial report for the insurance companies to avoid a selection bias [17].

Preoperative bowel preparation, placement of drains, creation of ileostomy or postoperative participation in an enhanced recovery programme were scheduled by the responsible department or surgeon. Anastomotic leak was diagnosed at the discretion of the providing surgeon whether by clinical and/or radiological means.

Exclusion criteria were (a) cancer localized higher than 16cm from the anal verge, (b) anal carcinoma, (c) treatment outside of Germany, (d) tumour stage UICC IV.

Demographic data included age, gender, body mass index (BMI - <25 kg/m^2^ vs. >25 kg/m^2^), American Society of Anaesthesiologists – Classification[18] (ASA) I+II vs. III+IV, smoking and alcohol history, diabetes.

Surgical course included type of anastomosis (stapled vs. hand suture), intraoperative blood transfusion, use of protective ileostomy, length of hospital stay, hospital mortality. For estimation of volume effect hospitals were classified into four groups based on quadrilles of patients treated annually (<14 patients per year (ppy), 15–24 ppy, 25–36 ppy, >36 ppy). Tumour stage was classified according to the UICC-classification. UICC stage was based on classification by the local pathologist. Patients with UICC stage IV were excluded. Tumour height was given as distance from the anal verge to the lower tumour border measured in rigid rectoscopy. The localization was then classified as lower rectum (0–6 cm), middle rectum (6–12 cm) and upper rectum (12–16 cm). Adjuvant treatment complied with the German S3-guidelines [19], but was left at the discretion of the responsible surgeon, oncologist or general practitioner. Follow up was based on the information received from family physicians, responsible for postoperative care in the German health system. Data were collected using a structured form provided by the Institute for Quality Assurance in Operative Medicine, Otto-von-Guericke-University, Magdeburg, Germany. Follow up was conducted annually. When available, data from local tumour registers were cross-checked.

### Statistical analysis

All constant variables were used with appropriate measurements and given as mean with standard deviation, minimum and maximum or as median with 25^th^ to 75^th^ percentile, minimum and maximum. Categorical variables were displayed as absolute or relative frequencies. Chi-square test was used to proof independency of categorical variables. For small sample numbers (<5) Cross-tabulation or Fisher's exact test were used. For estimation of systematic differences between groups test of normal distribution was performed (Shapiro-Wilk-test). In case of normal distribution of variables *T*-test was used and for non-even distribution of data *U*-test. Furthermore data were calculated for probability of survival according to Kaplan-Meier-model. Groups were compared using log-rank-testing in relation to survival. Additionally median survival with 95% confidence interval was calculated.

Risk factor analysis for occurrence of anastomotic leakage was first performed univariate. All statistical significant variables were further calculated using a multivariate regression and displayed as odds ratio with 95%-confidence interval. Significance was considered if *p* < 0.05. Statistical analysis was performed with IBM^®^ SPSS^®^ Statistics, Version 21.0.0, SPSS Inc. (New York, USA).

This study was approved by the local ethics committee and was undertaken with the understanding and appropriate informed consent of each patient included. Written consent was obtained from each patient.

## CONCLUSION

With 11.9% AL remains a common and serious complication of curative surgery in rectal cancer. In multivariate analysis male gender, ASA-classification ≥III, smoking history, alcohol history, intraoperative blood transfusion, no protective ileostomy, UICC-stage and height of tumour were independent risk factors. The majority of these factors is patient- and tumour-dependent and cannot be influenced by the surgeon. Only creation of a protective stoma as well as intraoperative blood transfusion remain at the surgeon's discretion. Anastomotic leakage does limit overall survival and disease free survival, but this difference is generated during the initial hospital stay. Despite the efforts of minimising the risk of AL, early detection and successful treatment of this complication (intensive care, interventional radiology etc.) seem to be crucial for preventing the negative influence on survival. This could support selection of high-risk patients (male, advanced tumours, ASA III-IV, smoking and/or alcohol history) for treatment in hospitals providing these services.
